# Clinical Utility of Broad-Panel Profiling in Non-Small Cell Lung Cancer: Pairwise Co-Mutational Gene Patterns and PD-L1 Associations Define Stage-Specific Prognosis

**DOI:** 10.3390/genes17101233

**Published:** 2026-10-05

**Authors:** Carolina Sassorossi, Elisa De Paolis, Jessica Evangelista, Davide Dalfovo, Alessandra Cancellieri, Ilenia Carriero, Annalisa Campanella, Esther G. C. Troost, Steffen Löck, Róza Ádány, Núria Farré, Ece Öztürk, Rocco Trisolini, Luca Boldrini, Alessio Stefani, Andrea Urbani, Emilio Bria, Stefano Margaritora, Angelo Minucci, Filippo Lococo

**Affiliations:** 1 Thoracic Surgery Unit, A. Gemelli University Hospital Foundation IRCCS, 00168 Rome, Italyfilippo.lococo@policlinicogemelli.it (F.L.); 2 Departmental Unit of Molecular and Genomic Diagnostics, Genomics Research Core Facility, Gemelli Science and Technology Park (GSTeP), Fondazione Policlinico Universitario A. Gemelli IRCCS, 00168 Rome, Italy; 3 Clinical Chemistry, Biochemistry and Molecular Biology Operations (UOC), Fondazione Policlinico Universitario A. Gemelli IRCCS, 00168 Rome, Italy; 4 OncoRay—National Center for Radiation Research in Oncology, Faculty of Medicine and University Hospital Carl Gustav Carus, TUD Dresden University of Technology, Helmholtz-Zentrum Dresden-Rossendorf, 01307 Dresden, Germany; 5 Institute of Pathology, A. Gemelli University Hospital Foundation IRCCS, 00168 Rome, Italy; 6 Department of Radiotherapy and Radiation Oncology, Faculty of Medicine and Universit Hospital Carl Gustav Carus, TUD Dresden University of Technology, 01307 Dresden, Germany; 7 ELKH-DE Public Health Research Group, Department of Public Health and Epidemiology, Faculty of Medicine, University of Debrecen, 4028 Debrecen, Hungary; 8 Institut de Recerca de l’Hospital de la Santa Creu i Sant Pau (IR-HSCSP), 08041 Barcelona, Spain; nfarre@santpau.cat; 9 School of Medicine, Turkey and Koç University Research Center for Translational Medicine (KUTTAM), Koç University, Sariyer, 34450 Istanbul, Turkey; 10 Interventional Pulmonology Unit, A. Gemelli University Hospital Foundation IRCCS, Largo A. Gemelli 8, 00168 Rome, Italy; 11 Dipartimento di Diagnostica per Immagini, Radioterapia Oncologica ed Ematologia, Fondazione Policlinico Universitario “A. Gemelli” IRCCS, 00168 Rome, Italy; 12 Istituto di Radiologia, Università Cattolica del Sacro Cuore, 00168 Rome, Italy; 13 Radiomics Core Research Facility, Gemelli Science and Technology Park (GSTeP), Fondazione Policlinico Universitario A. Gemelli IRCCS, 00168 Rome, Italy; 14 Medical Oncology, Comprehensive Cancer Center, IRCCS Fondazione Policlinico Universitario Agostino Gemelli, Largo Francesco Vito, 1, 00168 Rome, Italy; 15 Medical Oncology Unit, Ospedale Isola Tiberina-Gemelli Isola, 00168 Rome, Italy

**Keywords:** NSCLC, gene mutations, PD-L1

## Abstract

Objectives: Precision medicine has improved outcomes in advanced lung cancer, but its implementation remains limited. The prospective LANTERN project (NCT05802771) involves comprehensive clinical and multi-omics data collection. Here, we report preliminary genomic findings, focusing on the prognostic impact of gene mutations, pairwise co-mutational patterns, and their stage-specific association with PD-L1 analysis on patients speciments. Methods: This prospective, multicenter observational study enrolled 271 consecutive patients between March 2023 and December 2024, including 137 with stage I–III and 134 with stage IV disease. Comprehensive genomic profiling was performed using the TruSight Oncology 500 High Throughput assay to assess single nucleotide variants, insertions/deletions, copy number alterations, tumor mutational burden and microsatellite instability. The prognostic role of mutations and co-mutations was evaluated, together with associations between genomic alterations and PD-L1 expression. Results: At least one Tier I–II alteration was identified in 84% of patients. TP53 (39%), KRAS (27%), EGFR (15%), and STK11 (11%) were the most frequently altered genes. Several co-mutations were associated with worse overall survival, including TP53/STK11, STK11/LRP1B, STK11/RB1 and RB1/LRP1B, and with reduced disease-free survival, particularly TP53/KRAS, TP53/STK11, KRAS/STK11 and STK11/LRP1B. In advanced disease, STK11 and KEAP1 mutations were associated with low PD-L1 expression, whereas PIK3CA, PTEN and ARID1A mutations correlated with higher PD-L1 expression in early-stage tumors. Conclusions: Comprehensive genomic profiling reveals substantial molecular heterogeneity across lung cancer stages and identifies co-mutational patterns with prognostic and immune-related relevance, supporting broader molecular testing at diagnosis.

## 1. Introduction

Lung cancer remains the leading cause of cancer death worldwide and continues to carry a poor overall prognosis despite substantial advances in systemic therapy [1]. It is a highly heterogeneous neoplasm in which distinct histological subgroups have been clearly identified, each characterized by specific morphological features and molecular characteristics [2]. Among these, non-small cell lung cancer (NSCLC) accounts for approximately 85% of all lung tumors, with adenocarcinoma (ADC) representing its most frequent histological subtype, comprising more than 60% of NSCLC cases. Approximately 30% of patients with NSCLC present with surgically resectable disease (stages I–IIIA) and generally have a more favorable prognosis than those diagnosed with advanced disease. Over the past decade, the treatment of NSCLC has been reshaped by the arrival of targeted agents and immune checkpoint inhibitors, which have meaningfully extended survival in selected subgroups of patients [3]. As a result, molecular analysis is now routinely performed in advanced disease, allowing clinicians to identify actionable alterations and biomarkers capable of guiding therapy [4]. These targeted and immune-based strategies are also increasingly being introduced in the neoadjuvant and adjuvant contexts, making genomic profiling at diagnosis increasingly relevant, even for patients with early-stage disease [5]. While the clinical value of molecular alterations in guiding treatment selection is now well established, their value as prognostic markers remains far less defined, especially in real-world unselected NSCLC populations [6]. A growing body of literature has examined the association between genomic alterations and long-term clinical outcomes, including recurrence and survival. However, the available evidence remains heterogeneous, reflecting differences in patient populations, disease stage, sequencing platforms, and therapeutic strategies [7,8,9,10]. Consequently, the prognostic significance of the genomic landscape of NSCLC has yet to be fully established. The widespread adoption of high-throughput sequencing and comprehensive genomic profiling (CGP) provides an opportunity to address this knowledge gap. CGP has enabled the identification of a broad range of molecular alterations and dysregulated signaling pathways, leading to the definition of distinct genomic subtypes within NSCLC [4]. However, most studies to date have relied on targeted testing of individual driver mutations or have focused on cohorts enriched for advanced-stage disease, limiting the generalizability of their findings [11,12,13,14]. Prospective studies applying CGP across early- and locally advanced-stage NSCLC are therefore still needed to clarify the true prognostic impact of the molecular landscape and to support its routine incorporation into the risk stratification and into overall clinical patient management. In this framework, the LANTERN project [15] (Lung cancer multi-omics digital human avatars for integrating precision medicine) was conceived, with the aim of establishing a new paradigm for lung cancer decision-making through predictive digital platforms built on the integration of prospective complex, multi-omics data coming from a European multicentric consortium.

In the present analysis, in a large prospective real-world cohort of NSCLC patients, we investigated the prognostic role of individual and co-occurring genomic alterations and their association with PD-L1 expression detected in our cohort of patients, with the aim of evaluating the potential prognostic impact of this approach.

## 2. Materials and Methods

### 2.1. Ethics

The study protocol was developed in accordance with the principles of Good Clinical Practice of the European Union and the current revision of the Declaration of Helsinki. The study was approved by the Ethics Committee of Fondazione Policlinico Universitario Agostino Gemelli IRCCS—Università Cattolica del Sacro Cuore (Approval No. 5420–0002485/23) and registered at ClinicalTrials.gov (NCT05802771). All procedures were performed in compliance with applicable guidelines and regulations. Written informed consent was obtained from all participants or, when appropriate, from their legal representatives.

### 2.2. Patients

This prospective, multicenter observational study included 271 patients with non-small cell lung cancer (NSCLC) enrolled within the LANTERN consortium between March 2023 and December 2024 [15]. To ensure a representative cohort and minimize selection bias, enrollment was not restricted according to sex or disease stage.

Eligible patients were aged 18 years or older, had histologically confirmed NSCLC, and provided written informed consent. Exclusion criteria included a diagnosis of neuroendocrine carcinoma, small cell lung cancer, or other non-NSCLC pulmonary malignancies, as well as diagnoses established exclusively on cytological specimens.

Tumor staging was assigned according to the eighth edition of the American Joint Committee on Cancer (AJCC) TNM classification for lung cancer [16]. Clinicopathological information, including age, sex, smoking history, tumor location, histopathological characteristics, PD-L1 expression, treatment, and pathological TNM stage, was retrieved from electronic medical records.

Clinical and molecular data were prospectively collected and managed using the REDCap (Research Electronic Data Capture) platform hosted at Fondazione Policlinico Universitario A. Gemelli IRCCS. To protect patient confidentiality, all records were pseudonymized and linked through unique alphanumeric study identifiers.

### 2.3. Molecular Analysis

All patients with adequate tissue available for next-generation sequencing (NGS) underwent comprehensive molecular profiling regardless of tumor histology or disease stage. Hematoxylin- and eosin-stained slides were independently reviewed by experienced pathologists to identify representative tumor areas containing at least 20% viable tumor cells.

Genomic DNA was extracted from formalin-fixed, paraffin-embedded (FFPE) tissue using the AllPrep^®^ DNA/RNA FFPE Kit (QIAGEN^®^) (Venlo, The Netherlands/Hilden, Germany) according to the manufacturer’s protocol.

Comprehensive genomic profiling was performed using the TruSight Oncology 500 High Throughput (TSO500HT) assay (Illumina, San Diego, CA, USA), enabling the detection of single nucleotide variants (SNVs), insertions/deletions (indels), copy number variations (CNVs), tumor mutational burden (TMB), and microsatellite instability (MSI) across 523 cancer-related genes. Library preparation and sequencing were carried out following the manufacturer’s recommendations and previously validated protocols.

Sequencing data were analyzed using the VELsera Clinical Genomics Workspace (Cloud-based SaaS (Software-as-a-Service), integrating information from curated molecular knowledge bases, clinical practice guidelines, FDA-approved therapeutic indications, and ongoing clinical trials. Genetic variants were classified according to the joint recommendations of the Association for Molecular Pathology, the American Society of Clinical Oncology, and the College of American Pathologists [17].

### 2.4. PD-L1 Testing

PD-L1 immunohistochemistry (IHC) was performed using clone SP263 on the Ventana benchmark platform (Tucson/Oro Valley, AZ, USA) according to the manufacturer’s protocol. Positive control was used on each slide. Scoring was based on tumor cell membrane staining of any intensity, and a minimum of 100 tumor cells was required to render a recorded score. The tumor proportion score (TPS) was calculated as the number of tumor cells showing complete or incomplete membranous staining divided by the total number of viable tumor cells, multiplied by 100. PD-L1 subgroups were defined as negative (PD-L1 < 1%), intermediate (PD-L1 1–49%), and high (PD-L1 ≥ 50%). PD-L1 evaluation was available in 229 of 271 cases due to (1) NOS lung tumors (10 cases, see above) and (2) limited tissue from biopsy (32 cases).

### 2.5. Statistical Analysis

All statistical analyses were performed using R software (version 4.3). Continuous variables, including age and tumor mutational burden (TMB), were summarized as medians with ranges, whereas categorical variables—including sex, smoking status, disease stage, performance status, and PD-L1 expression—were reported as frequencies and percentages. Somatic mutation data were analyzed using the maftools R package 4. For selected clinically relevant biomarkers and Tier I–II variants, the frequencies of single nucleotide variants, insertions/deletions, and copy number alterations were calculated for the entire cohort and subsequently compared across disease stages. Fisher’s exact test was applied to evaluate both overall and stage-specific enrichment of genomic alterations, as well as associations between key driver mutations (including *TP53*, *KRAS*, and *EGFR*) and adenocarcinoma growth patterns.

Overall survival (OS) and disease-free survival (DFS) were analyzed using multivariable Cox proportional hazards regression models adjusted for pathological stage. OS was defined as the interval from diagnosis to death from any cause, with patients alive at last follow-up censored on that date. DFS was evaluated only in patients with stage I–III disease after curative treatment and calculated from treatment to disease recurrence, death, or last follow-up. Hazard ratios (HRs) with 95% confidence intervals (CIs) were reported.

Separate Cox regression models were fitted to evaluate: (i) the prognostic impact of individual gene alterations; (ii) pairwise co-mutation profiles; (iii) clinical variables; and (iv) combined gene–clinical interaction variables. For co-mutation analyses, patients were categorized according to the mutational status of each gene pair (wild-type, single-gene mutation, or co-mutation), using the double wild-type group as the reference category. Combined gene–clinical variables were generated by integrating mutation status with selected clinicopathological factors (including ECOG performance status, smoking status, body mass index, pathological score, treatment exposure, and tumor size), and their associations with survival outcomes were evaluated in separate multivariable models.

Given the exploratory nature of this prospective observational analysis, no formal a priori power calculation was performed for each individual gene or co-mutational subgroup. The overall cohort included 271 patients, allowing detection of relatively large effects for common alterations but limiting statistical power for rare variants and low-frequency co-mutational profiles. Therefore, co-mutational analyses were restricted to predefined pairwise mutation-status profiles rather than high-dimensional network inference or complex stage-dependent interaction modeling. Results from rare subgroups were interpreted cautiously, with subgroup sizes, event numbers, HRs, 95% CIs, nominal *p*-values, and FDR-adjusted q-values reported to support transparent interpretation.

## 3. Results

### 3.1. Patient Cohort

The study cohort comprised 271 patients with NSCLC (Table 1), with a balanced sex distribution (49.8% female and 50.2% male) and a median age of 69 years (range, 28–87 years). At diagnosis, 35.8% of patients presented with early-stage disease, 14.8% with locally advanced disease, and 49.5% with metastatic disease (stage IV).

Adenocarcinoma represented the predominant histological subtype (89.7%), whereas squamous cell carcinoma accounted for 7.3% of cases. Mixed histologies were infrequent, together comprising less than 3% of the cohort. The median tumor mutational burden (TMB) was 6.28 mutations/Mb (range, 0–94.3), with low, intermediate, and high TMB observed in 42.0%, 31.5%, and 26.5% of tumors, respectively.

PD-L1 expression was available for 229 patients. Overall, 34.1% (78/229) of tumors were PD-L1 negative (<1%), 28.8% (66/229) showed intermediate expression (1–49%), and 37.1% (85/229) exhibited high PD-L1 expression (≥50%).

### 3.2. Distribution of the Most Frequently Mutated Genes

**Figure 1:**
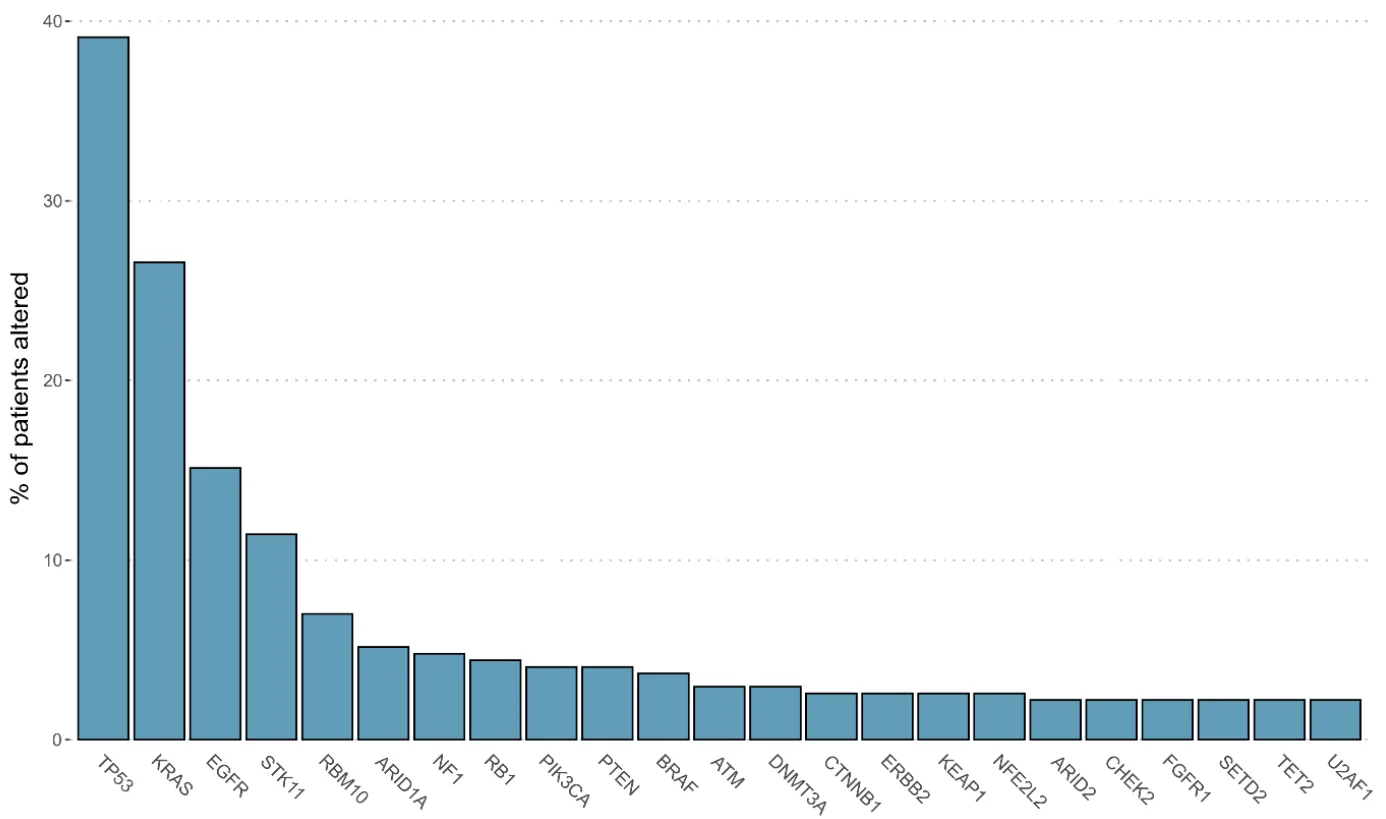
Genomic landscape and distribution of somatic mutations (Tier I-II, SNVs, indels). Bar chart representing the frequency of the most prevalent genetic alterations identified [18].

At least one Tier I–II molecular alteration was identified in 84% of patients. The distribution of the most frequently mutated genes revealed that *TP53* was the most commonly altered gene, with mutations detected in approximately 39% of patients. *KRAS* was the second most frequently mutated gene (approximately 27%), followed by *EGFR* (15%) and *STK11* (11%). Mutations in *RBM10* were observed in around 7% of cases, whereas alterations in *ARID1A*, *NF1*, *RB1*, *PIK3CA*, *PTEN*, and *BRAF* occurred at frequencies ranging from 4% to5%. The remaining genes, including *ATM*, *DNMT3A*, *CTNNB1*, *ERBB2*, *KEAP1*, *NFE2L2*, *ARID2*, *CHEK2*, *FGFR1*, *SETD2*, *TET2*, and *U2AF1*, were each mutated in approximately 2–3% of patients, indicating a long tail of less frequently occurring genomic alterations (Figure 1, from a previous study of Sassorossi et al. [18]).

### 3.3. Prognostic Impact of Genomic Alterations, Co-Mutations, and Clinical Variables

#### 3.3.1. Single-Gene Analysis

**Figure 2:**
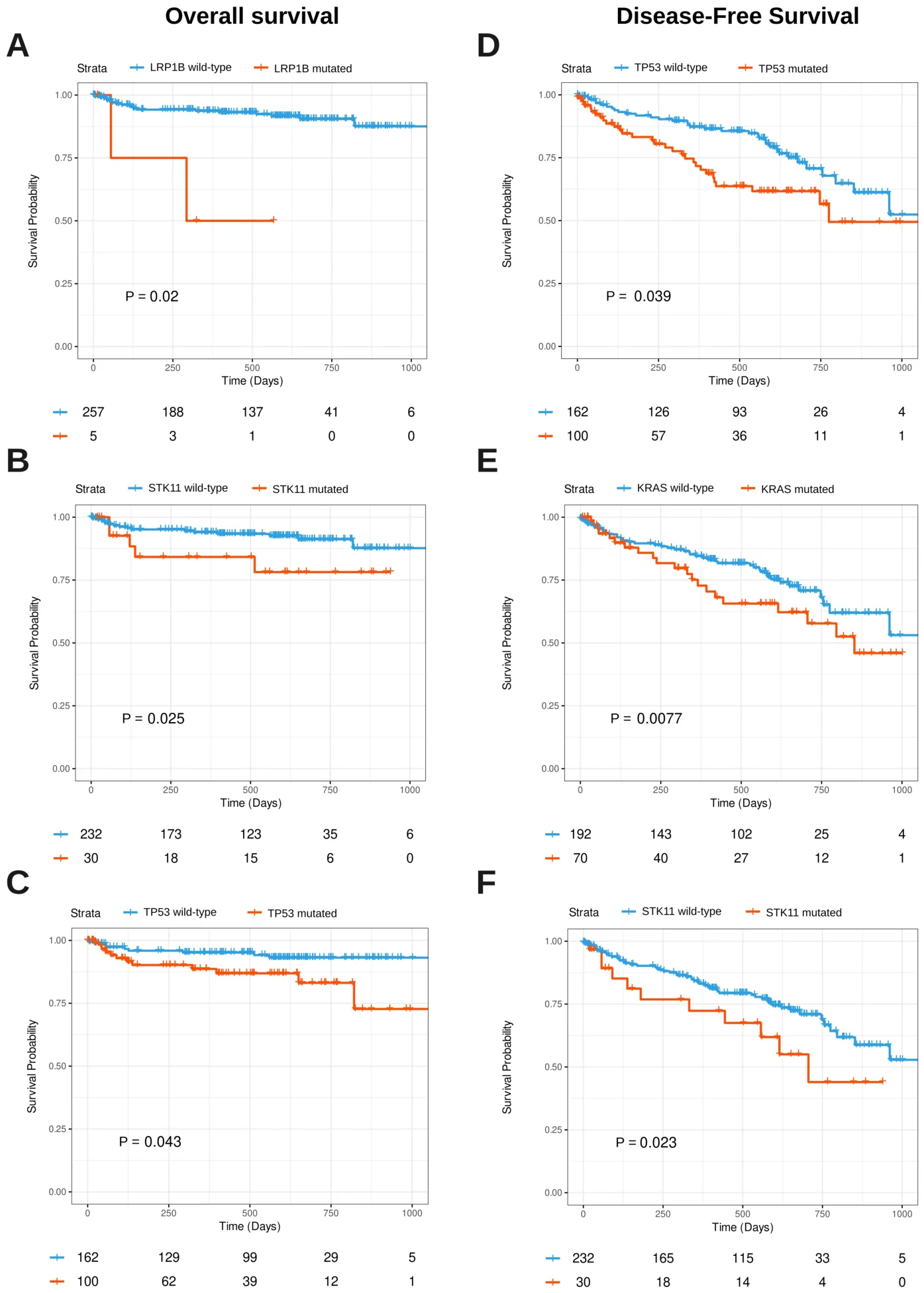
Prognostic impact of selected single-gene alterations on overall survival and disease-free survival. (**A**–**C**) Kaplan–Meier estimates of overall survival (OS) according to the mutational status of LRP1B (**A**), STK11 (**B**), and TP53 (**C**). (**D**–**F**) Kaplan–Meier estimates of disease-free survival (DFS) according to the mutational status of TP53 (**D**), KRAS (**E**), and STK11 (**F**). In all panels, the mutated cohorts are compared against their respective wild-type reference groups. Nominal two-sided *p*-values were derived from multivariable Cox proportional hazards regression models adjusted for pathological stage.

Multivariable Cox regression analyses adjusted for pathological stage identified several individual genomic alterations showing nominal associations with survival outcomes. For overall survival (OS), mutations in LRP1B (Figure 2A), STK11 (Figure 2B), and TP53 (Figure 2C) were associated with an increased risk of death, with LRP1B showing the strongest adverse prognostic effect. Similarly, for disease-free survival (DFS), mutations in TP53 (Figure 2D), KRAS (Figure 2E), and STK11 (Figure 2F) showed nominal associations with an increased risk of recurrence, confirming their prognostic relevance in the study cohort. Subgroup sizes, event numbers, HRs, 95% CIs, nominal *p*-values, and FDR-adjusted q-values are reported in Table 2. After Benjamini–Hochberg FDR correction, none of the reported associations retained formal significance at q < 0.05. However, all associations highlighted in the main text showed FDR q-values below 0.25 and were therefore interpreted as exploratory clinical-genomic signals, prioritized according to effect size, biological relevance, and consistency with the study objectives.

#### 3.3.2. Co-Mutation Analysis

The evaluation of co-occurring genomic alterations revealed that several mutation combinations conferred an even poorer prognosis than single-gene alterations alone. For OS, co-mutations involving *TP53/STK11* (*p* = 0.018), *STK11/LRP1B* (*p* = 0.003), *STK11/RB1* (*p* = 0.013), and *RB1/LRP1B* (*p* = 0.013) were nominally associated with worse survival (Table 3 and Figure S1). Likewise, for DFS, multiple co-mutational profiles—including *TP53/KRAS* (*p* = 0.0032), *TP53/STK11* (*p* = 0.0096), *KRAS/STK11* (*p* = 0.023), *STK11/RB1* (*p* = 0.036), *STK11/PTEN* (*p* = 0.046), *STK11/ATM* (*p* = 0.0093), and *STK11/LRP1B* (*p* = 0.015)—were associated with a nominally increased risk of disease recurrence, highlighting the cumulative prognostic impact of concurrent genomic alterations (Table 3 and Figure S2).

#### 3.3.3. Integrated Gene–Clinical Analysis

To further investigate the interaction between molecular and clinical variables, combined gene–clinical models were generated. These analyses demonstrated that the prognostic effect of specific genomic alterations was influenced by the clinical context. In particular, patients harboring *STK11* mutations with ECOG > 1 experienced the poorest OS (*p* = 0.028), whereas targeted therapy was associated with improved survival in the *TP53*-mutated subgroup (*p* = 0.005) and in patients with *EGFR* mutations (*p* = 0.006). However, because TP53 is not an actionable driver in current NSCLC practice, this finding should be interpreted as a treatment-context association rather than as evidence of a direct TP53-mediated therapeutic benefit. Additional multivariable Cox regression models including pathological stage, smoking status, and tumor mutational burden were fitted for *TP53* and *LRP1B*. After covariate adjustment, *TP53* mutation status was no longer associated with survival, whereas *LRP1B* mutation retained an adverse association with overall survival (Figure S2 and Table S1).

#### 3.3.4. PD-L1 Expression and Mutational Correlations

**Figure 3:**
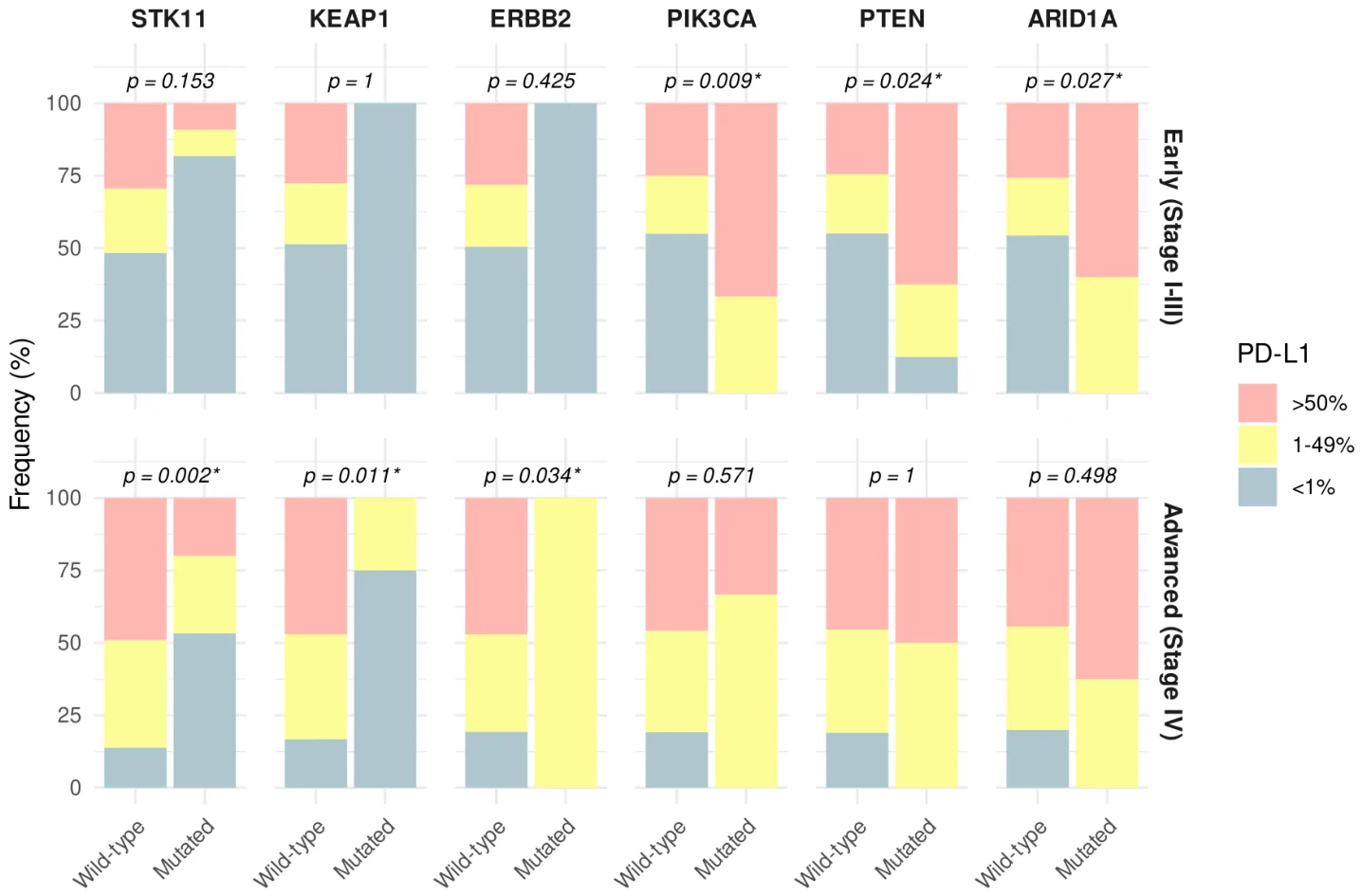
PD-L1 expression according to mutational status and disease stage. Stacked bar plots showing the distribution of PD-L1 expression (<1%, 1–49%, and ≥50%) in wild-type and mutated tumors for STK11, KEAP1, ERBB2, PIK3CA, PTEN, and ARID1A, stratified by early-stage (I–III) and advanced-stage (IV) disease. *p*-values indicate comparisons between wild-type and mutated groups. * *p*-value reached significance.

PD-L1 expression was evaluated according to the mutational status of selected genes and stratified by disease stage (early-stage I–III vs. advanced-stage IV) (Figure 3). In early-stage tumors, mutations in *PIK3CA* (*p* = 0.009), *PTEN* (*p* = 0.024), and *ARID1A* (*p* = 0.027) were significantly associated with higher PD-L1 expression, characterized by an increased proportion of tumors with PD-L1 ≥ 1% and, particularly, >50%, compared with wild-type cases. No significant differences in PD-L1 distribution were observed according to *STK11*, *KEAP1*, or *ERBB2* mutational status (*p* > 0.05). In contrast, among patients with advanced-stage disease, mutations in *STK11* (*p* = 0.002), *KEAP1* (*p* = 0.011), and *ERBB2* (*p* = 0.034) were significantly associated with a shift toward lower PD-L1 expression, with a higher proportion of PD-L1-negative tumors (<1%) compared with wild-type tumors. No significant associations were detected for *PIK3CA*, *PTEN*, or *ARID1A* mutations in advanced-stage disease (*p* > 0.05). These findings suggest that the relationship between specific genomic alterations and PD-L1 expression differs according to disease stage.

## 4. Discussion

In this study, we comprehensively evaluated the prognostic impact of genomic alterations, co-mutation patterns, and clinical characteristics in patients with NSCLC. Our findings demonstrate that specific somatic mutations, particularly in *STK11*, *TP53*, *KRAS*, and *LRP1B*, are independently associated with poorer survival outcomes, while combinations of these alterations identify subgroups with an even more unfavorable prognosis. These results support the growing evidence that the genomic landscape, rather than single driver mutations alone, contributes substantially to the biological heterogeneity and clinical behavior of NSCLC [19].

### 4.1. Key Prognostic Findings and Co-Mutation Synergy

Among individual alterations identified in the cohort of analyzed patients, *STK11* emerged as one of the strongest and most consistent prognostic biomarkers, being independently associated with both shorter overall and disease-free survival. This direct observation is in agreement with previous studies showing that *STK11* loss promotes tumor progression, metabolic reprogramming, and resistance to antitumor immune responses, ultimately resulting in more aggressive disease [20]. Additionally, in the literature *STK11* loss has been linked to increased tumor aggressiveness, metabolic rewiring, and the development of an immune-suppressed (“cold”) tumor microenvironment, characterized by reduced T-cell infiltration and diminished responsiveness to immune checkpoint inhibitors [21].

Moreover, the particularly poor outcomes observed in tumors harboring *STK11* co-mutations, especially in combination with *TP53* or *KRAS*, are in line with previous genomic studies demonstrating that co-occurring alterations define biologically distinct molecular subgroups with different clinical behaviors and therapeutic vulnerabilities [22].

Likewise, *TP53* mutations were associated with inferior OS and DFS, confirming their established role as markers of genomic instability and poor prognosis [23]. *KRAS* mutations independently predicted disease recurrence, consistent with their known association with aggressive tumor biology [24], while *LRP1B* mutations exhibited the strongest adverse effect on overall survival. To note, due to the small number of patients harboring this specific gene alteration, the observed result must be interpreted with caution. Although *LRP1B* has previously often been linked to smoking-related mutational processes, increased tumor mutational burden and enhanced response to immune checkpoint inhibitors, its independent association with poor survival is retained after adjustment for covariates. In our cohort, the persistence of the LRP1B survival association after adjustment suggests that its prognostic effect was not solely explained by smoking exposure or TMB. Nevertheless, because only a small number of patients harbored LRP1B mutations, this finding should be regarded as exploratory and requires validation in larger independent cohorts.

Importantly, our co-mutation analysis demonstrated that concurrent genomic alterations provided greater prognostic discrimination than individual mutations alone. In particular, combinations involving *STK11*, including *TP53/STK11*, *KRAS/STK11*, *STK11/LRP1B*, and *STK11/RB1*, were associated with markedly worse survival outcomes [25]. These findings are biologically plausible, as *STK11* loss has been shown to cooperate with alterations affecting cell-cycle regulation, DNA damage repair, and tumor suppressor pathways, resulting in enhanced tumor aggressiveness and therapeutic resistance [21].

The markedly worse survival observed in patients with both *STK11* mutations and impaired performance status (ECOG ≥ 1) supports the concept that molecular and host-related factors synergistically contribute to disease aggressiveness. *STK11* inactivation has consistently been associated with poor prognosis, immune-resistant tumor biology, and inferior outcomes across treatment modalities, and our findings indicate that this adverse molecular profile may be further amplified in clinically frail patients [20,21]. Conversely, the apparent survival benefit of targeted therapy among patients harboring *TP53* mutations is consistent with the accumulating evidence that the prognostic significance of *TP53* alterations is context-dependent, varying according to co-occurring genomic events, clinical variables, and treatment strategy. However, this association was not retained in additional multivariable models incorporating smoking status and TMB. Therefore, TP53 should be interpreted in our cohort as a marker of genomic complexity and treatment context rather than as an independent prognostic determinant. This distinction is clinically relevant because TP53 is not itself a targetable driver in current NSCLC practice, and apparent treatment-related associations in TP53-mutated tumors may reflect co-occurring actionable alterations, smoking-associated biology, disease stage, or treatment-selection effects rather than a direct TP53-mediated therapeutic benefit.

### 4.2. Stage-Dependent Genomic-Immune Discordance

Another relevant finding was the stage-dependent association between genomic alterations and PD-L1 expression. In early-stage disease, *PIK3CA*, *PTEN*, and *ARID1A* mutations were associated with higher PD-L1 expression, whereas in advanced-stage tumors, *STK11*, *KEAP1*, and *ERBB2* mutations correlated with lower PD-L1 levels [26,27]. The association between *STK11* and *KEAP1* mutations and reduced PD-L1 expression is consistent with previous reports describing these alterations as markers of an immune-cold phenotype and reduced responsiveness to immune checkpoint inhibitors.

### 4.3. Clinical Translation and Future Directions

Our findings underscore the pivotal role of integrating comprehensive genomic profiling (CGP) into routine clinical decision-making from the early stages of non-small cell lung cancer (NSCLC). Rather than relying solely on individual driver mutations or single-gene testing, evaluating co-mutational landscapes—specifically involving *STK11*, *TP53*, *KRAS*, and *LRP1B*—provides superior prognostic stratification and identifies distinct biological subgroups with heightened recurrence risk and adverse overall survival. Crucially, the observed stage-dependent discordance between genomic alterations and PD-L1 expression (e.g., *PIK3CA/PTEN* enrichment in PD-L1-high early-stage tumors versus *STK11/KEAP1* enrichment in PD-L1-negative advanced tumors) suggests a stage-specific association between genomic alterations and PD-L1 expression. Recognizing these genomic signatures at diagnosis empowers clinicians to tailor therapeutic strategies, intensify post-operative surveillance in high-risk patients, and optimize candidate selection for immune checkpoint blockade and targeted regimens.

#### Limitations and Points of Strength

This study presents several points of strength but also important limitations and biases. A major strength of this study lies in its prospective, real-world design incorporating an unselected multicenter cohort of NSCLC patients spanning early to advanced stages, evaluated with a broad 523-gene panel (TruSight Oncology 500 HT). Furthermore, the simultaneous characterization of single-gene alterations, pairwise co-mutation patterns, stage-stratified PD-L1 expression, and integrated gene–clinical models provides a highly granular mapping of disease heterogeneity. However, several limitations must be acknowledged. First, the treatment regimens received across stage-stratified subgroups were heterogeneous, potentially confounding long-term survival outcomes. Second, low sample sizes in specific rare co-mutation subsets (e.g., *LRP1B* mutations) led to wide confidence intervals for certain hazard ratios; for this reason, the role of *LRP1B* mutations in OS may be incidental and only due to the small number of patients with this kind of mutation. Finally, the absence of an independent external validation cohort and the use of nominal *p*-values without strict multiple testing corrections necessitate cautious interpretation and warrant prospective validation in larger multi-institutional cohorts.

## 5. Conclusions

Our findings indicate that *STK11*, *TP53*, *KRAS*, and *LRP1B* mutations, particularly when occurring in combination, identify patients with significantly worse clinical outcomes. Furthermore, the differential association between genomic alterations and PD-L1 expression according to disease stage highlights the stage-specific association between genomic alterations and PD-L1 expression. These results support the incorporation of co-mutational profiling into prognostic models and provide additional rationale for integrating comprehensive genomic characterization into the clinical management of NSCLC.

## Figures and Tables

**Table 1:** Clinicopathological features.

	*N* = 271	%
Sex		
Female	135/271	49.82%
Male	136/271	50.18%
Stage		
Early (stage I)	97/271	35.79%
Locally Advanced (stage II–III)	40/271	14.76%
Advanced (stage IV)	134/271	49.45%
Kind of Histology Acquisition		
Surgery (anatomical lung resection)	137/271	50.5%
Surgery (non-anatomical lung resection)	25/271	9.2%
Biopsy (from primary or metastatic site)	109/271	40.3%
Age at Diagnosis		
Median (min–max)	69 (28–87)	
Smoker		
Yes	75/268	27.99%
Ex-smoker	126/268	47.01%
No	67/268	25%
PD-L1		
<1%	78/229	34.06%
1–49%	66/229	28.82%
>50%	85/229	37.12%
Histotype		
Adenocarcinoma	234/271	86.3%
Adenosquamous	2/271	0.74%
NEC°/NSCLC	6/271	2.2%
Squamous cell carcinoma	19/271	7.1%
NOS *	10/271	3.66%
TMB		
Median (min–max)	6.28 (0–94.3)	
§TMB Grade		
Low	92/219	42.01%
Medium	69/219	31.51%
High	58/219	26.48%

* NOS: NSCLC with no other specifies; ° NEC: neuroendocrine carcinoma; §TMB: tumor mutational burden.

**Table 2:** Multivariable Cox regression analyses of genomic mutation profiles associated with OS and DFS. Cox models were adjusted for pathological stage. Nominal two-sided *p*-values are shown. Adjusted q-values were calculated using the FDR method.

Endpoint	Gene	N Mutated	N Events	HR (95% CI)	*p*-Value	q-Value
OS	LRP1B	5	2	10.21 (2.29–45.58)	0.020	0.203
OS	STK11	30	5	3.76 (1.33–10.60)	0.025	0.203
OS	TP53	100	12	2.45 (1.02–5.85)	0.043	0.231
DFS	KRAS	70	21	2.17 (1.26–3.73)	0.007	0.123
DFS	STK11	30	11	2.33 (1.19–4.56)	0.023	0.187
DFS	TP53	100	30	1.69 (1.03–2.77)	0.039	0.206

**Table 3:** Multivariable Cox regression analyses of pairwise mutation-status profiles associated with OS and DFS. Cox models were adjusted for pathological stage. Nominal two-sided *p*-values are shown. Adjusted q-values were calculated using the FDR method.

Endpoint	Co-Occurrence	Reported Contrast	N (Profile/Ref)	Events (Profile/Ref)	HR (95% CI)	*p*-Value	q-Value
OS	TP53/STK11	Both Mut vs. Both WT	7/141	2/7	6.20 (1.79–32.62)	0.018	0.226
OS	STK11/LRP1B	Either Mut vs. Both WT	36/235	10/15	5.46 (2.11–14.15)	0.003	0.205
OS	STK11/RB1	Either Mut vs. Both WT	43/228	17/15	4.76 (1.83–12.35)	0.013	0.226
OS	RB1/LRP1B	Either Mut vs. Both WT	17/254	14/18	7.34 (2.37–22.71)	0.013	0.226
DFS	TP53/KRAS	Both Mut vs. Both WT	26/118	7/22	2.52 (1.49–4.27)	0.003	0.153
DFS	TP53/STK11	Both Mut vs. Both WT	7/139	3/28	3.26 (1.72–20.56)	0.010	0.174
DFS	KRAS/STK11	Both Mut vs. Both WT	17/179	8/42	2.66 (1.19–5.95)	0.023	0.215
DFS	STK11/RB1	Either Mut vs. Both WT	42/220	23/51	2.38 (1.28–4.43)	0.036	0.249
DFS	STK11/PTEN	Either Mut vs. Both WT	41/221	22/51	2.29 (1.22–4.28)	0.046	0.260
DFS	STK11/ATM	Either Mut vs. Both WT	38/224	19/52	2.74 (1.48–5.10)	0.009	0.174
DFS	STK11/LRP1B	Either Mut vs. Both WT	35/227	16/53	2.63 (1.40–4.95)	0.015	0.216

## Data Availability

The original contributions presented in this study are included in the article and Supplementary Materials. Further inquiries can be directed to the corresponding author.

## Supplementary Materials

The following supporting information can be downloaded at: https://www.mdpi.com/article/10.3390/genes17101233/s1. Figure S1. Pairwise mutation-status profiles associated with overall survival. Figure S2. Pairwise mutation-status profiles associated with disease-free survival and integrated gene–clinical overall survival profile. Table S1. Multivariable Cox regression analyses of integrated gene–clinical profiles and covariate-adjusted sensitivity models for TP53 and LRP1B.

## Abbreviations

The following abbreviations are used in this manuscript:

ADC	Adenocarcinoma
AJCC	American Joint Committee on Cancer
AI	Artificial Intelligence
CI	Confidence Interval
CGP	Comprehensive Genomic Profiling
CNV	Copy Number Variation
DFS	Disease-Free Survival
ECOG	Eastern Cooperative Oncology Group
FDA	Food and Drug Administration
FFPE	Formalin-Fixed, Paraffin-Embedded
HR	Hazard Ratio
IHC	Immunohistochemistry
indel	Insertion/Deletion
LC	Lung Cancer
LANTERN	Lung cancer multi-omics digital human avatars for integrating precision medicine into clinical practice
MSI	Microsatellite Instability
NEC	Neuroendocrine Carcinoma
NGS	Next-Generation Sequencing
NOS	Not Otherwise Specified
NSCLC	Non-Small Cell Lung Cancer
OS	Overall Survival
PD-1	Programmed Cell Death Protein 1
PD-L1	Programmed Death-Ligand 1
REDCap	Research Electronic Data Capture
SNV	Single Nucleotide Variant
TMB	Tumor Mutational Burden
TNM	Tumor, Node, Metastasis
TPS	Tumor Proportion Score
TSO500HT	TruSight Oncology 500 High-Throughput
