# Supplementary material for: Clinical Utility of Broad-Panel Profiling in Non-Small Cell Lung Cancer: Pairwise Co-Mutational Gene Patterns and PD-L1 Associations Define Stage-Specific Prognosis
Source: Genes (Basel). 2026 Oct 5;17(10):1233. doi: 10.3390/genes17101233 (PMC13704353; doi:10.3390/genes17101233)
Supplement: Supplementary file 1 [file genes-17-01233-s001.zip › genes-4542734-supplementary.pdf]

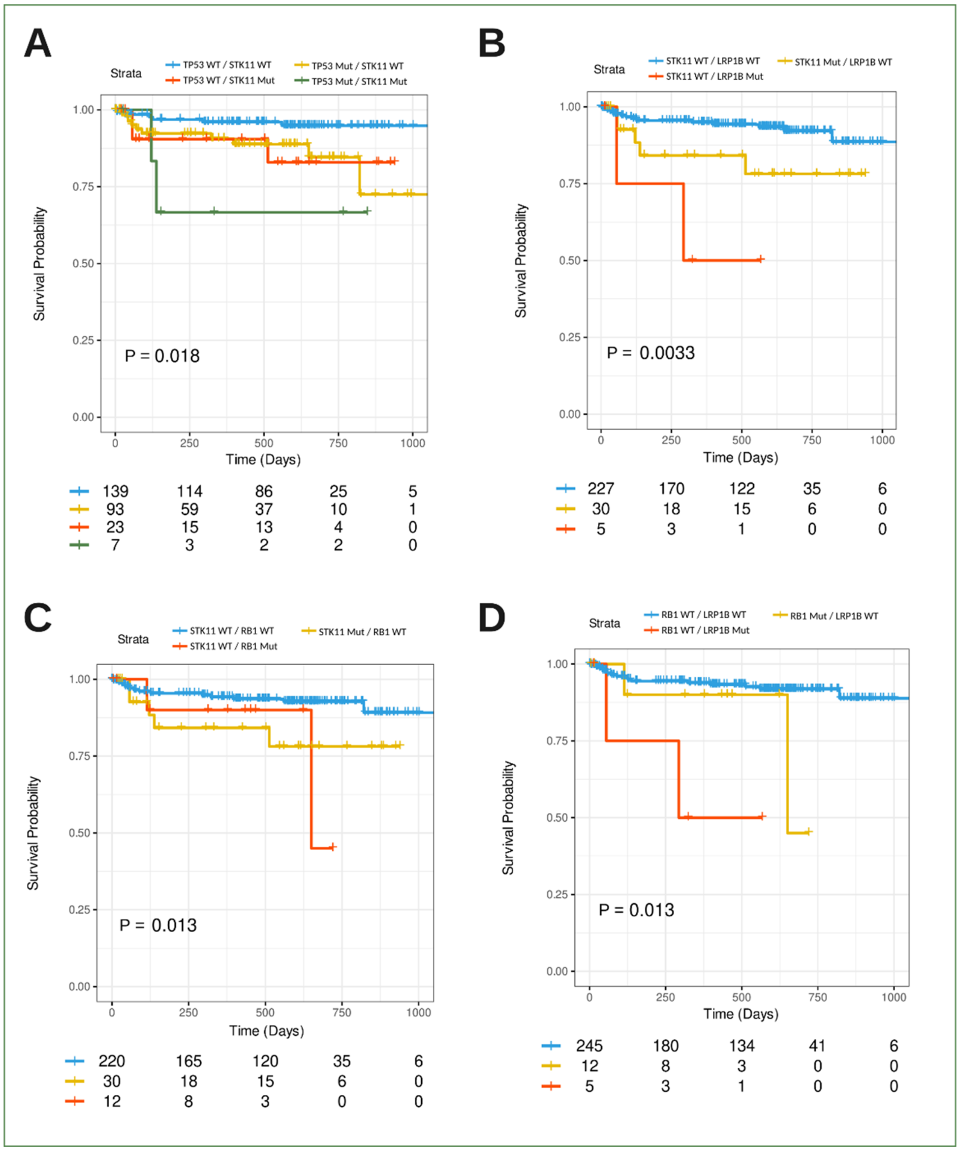

Supplementary Figure S1. Pairwise mutation-status profiles associated with overall survival. Kaplan–Meier estimates of overall survival (OS) according to selected pairwise gene mutation-status profiles. Panels show OS stratified by mutation status for **(A)** TP53/STK11, **(B)** STK11/LRP1B, **(C)** STK11/RB1, and **(D)** RB1/LRP1B. In all panels, the mutated cohorts are compared against their respective wild-type reference groups. Nominal two-sided P-values were derived from multivariable Cox proportional hazards regression models adjusted for pathological stage.

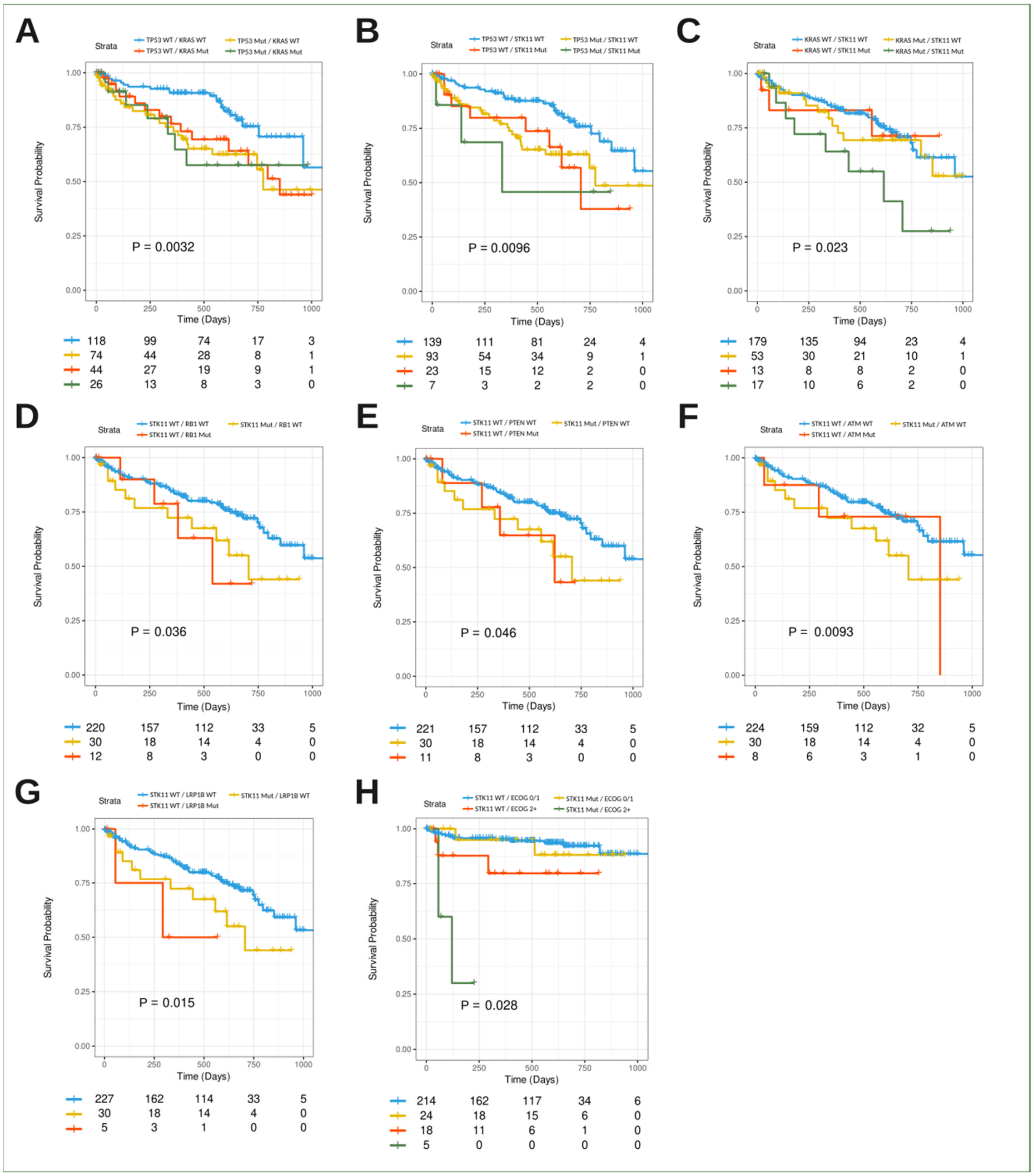

Supplementary Figure S2. Pairwise mutation-status profiles associated with disease-free survival and integrated gene–clinical overall survival profile. Kaplan–Meier estimates of disease-free survival (DFS) according to selected pairwise gene mutation-status profiles. Panels show DFS stratified by mutation status for **(A)** TP53/KRAS, **(B)** TP53/STK11, **(C)** KRAS/STK11, (**D**) STK11/RB1, **(E)** STK11/PTEN, **(F)** STK11/ATM, **(G)** STK11/LRP1B, and (**H**) Kaplan–Meier estimates of OS according to the integrated STK11 mutation/ECOG performance-status profile. In all panels, the mutated cohorts are compared against their respective wild-type reference groups. Nominal two-sided P-values were derived from multivariable Cox proportional hazards regression models adjusted for pathological stage.

Supplementary Table S1. Multivariable Cox regression analyses of integrated gene–clinical profiles and covariate-adjusted sensitivity models for TP53 and LRP1B. Nominal two-sided P values are shown. Adjusted q-values were calculated using the FDR method.

Endpoint	Co-occurrence	N (profile / ref)	Events (profile / ref)	HR (95% CI)	p-value	q-value
OS	STK11 / ECOG	5 / 214	3 / 14	9.74 (2.52-37.60)	0.028	0.081
OS	TP53/Target Therapy	93 / 35	53 / 8	0.42 (0.17-1.00)	0.005	0.038
OS	EGFR/Target Therapy	64 / 64	26 / 14	0.26 (0.10-0.65)	0.006	0.043
OS	TP53 + TMB + Smoking status	100	12	1.43 (0.49-4.16)	0.504	/
OS	LRP1B + TMB + Smoking status	5	2	10.05 (1.81-55.83)	0.027	/
